# Who chooses to enroll in a new national gambling self-exclusion system? A general population survey in Sweden

**DOI:** 10.1186/s12954-020-00423-x

**Published:** 2020-10-21

**Authors:** A. Håkansson, V. Henzel

**Affiliations:** 1 Department of Clinical Sciences Lund, Psychiatry, Faculty of Medicine, Lund University, Lund, Sweden; 2 Malmö Addiction Center, Region Skåne, Södra Förstadsgatan 35, plan 4, 205 02 Malmö, Sweden

**Keywords:** Gambling, Gambling disorder, Self-exclusion, Indebtedness, Gender

## Abstract

**Background:**

Self-exclusion from gambling is a common method for prevention and harm reduction in hazardous gambling. However, few national self-exclusion programs, involving a large number of gambling operators and activities in a country, have been assessed scientifically. This study aimed to examine characteristics of individuals who chose to enroll in a recently introduced (January, 2019) national self-exclusion system in Sweden.

**Methods:**

Adults and adolescents (from age 16 and above) were addressed with an online survey sent to members of the web panel of a market survey company (1940 respondents). Psychological distress, previous history of addictive disorders, sociodemographic data, and recent history of gambling patterns and over-indebtedness were recorded. Logistic regression tested associations with self-exclusion, with unadjusted analyses conducted for the sub-group of moderate-risk or problem gamblers.

**Results:**

Four percent reported having self-excluded using the new national self-exclusion system. In logistic regression, self-exclusion was significantly associated with younger age (OR 0.65 [0.54–0.79] for increasing age groups) and with the highest level of problem gambling (OR 2.84 [1.10–7.37]). In moderate-risk or problem gamblers, in unadjusted analyses, younger age (*p* < 0.05) and psychological distress (*p* = 0.02) were associated with self-exclusion. In none- or low-risk gamblers, 3% had self-excluded, which was significantly associated with younger age (*p* < 0.001) and self-reported over-indebtedness (*p* < 0.001).

**Conclusions:**

In a national, multi-venue online and land-based self-exclusion system, aiming to reduce the harm of problem gambling, self-exclusion is expectedly more common in problem gamblers, but also occurs among people without recent gambling problems. Further efforts may be needed in order to increase gambling self-exclusion in problem gamblers, and research in reasons for self-excluding, even in non-problem gamblers, is needed.

## Background

Problem gambling, including the sub-entity referred to as the gambling disorder diagnosis, affects a significant minority of adults worldwide, in most studies with a prevalence rate ranging between 1 and 5% of adults [1]. Among preventive and harm reduction strategies suggested for problem gambling, voluntary self-exclusion from gambling is an intervention suggested in previous research, with scientific reports emerging from the early twenty-first century [2–5]. In these programs, a person, assumingly with a problematic gambling behavior or a perceived risk of developing such a behavior, is able to voluntarily self-exclude from gambling, in order to prevent her/his own potential relapse into gambling in the future, during the time period defined at the moment of exclusion. However, the evidence of such measures has been described as limited in recent review papers, which call for further research in the area [6, 7]. Traditionally, self-exclusion programs have been related to physical gambling venues, typically casinos [2, 3, 8–10]. In land-based gambling, summarized findings have revealed at least promising effects post-exclusion compared to the pre-exclusion situation. A systematic review by Kotter and co-workers summarized that among self-excluders, a large majority report symptoms of psychological distress when they initiate self-exclusion, which indicates the need for such interventions. Also, the same review paper demonstrated that although the extent of improvement was very variable, included studies generally demonstrated lower rates of pathological gambling at follow-up than upon self-exclusion [11].

Less is known about self-exclusion in the online setting, and self-excluding from gambling was reported only by a minority of problem gamblers in a study in the online gambling modality [12]. In online gamblers in Australia, it has been reported that few gamblers use self-exclusion services such as time-out tools during gambling, but the use of the latter was unsurprisingly more common in problem gamblers than in gamblers with low-risk or non-risk gambling [13]. Caillon and co-workers reported data from a controlled intervention study in France, with an intervention consisting of a short-term temporary exclusion from separate online gambling sites. Although several outcome measures did not improve, cognitions related to gambling, and a sub-score derived from a craving measure, were improved at 2-month follow-up [14]. Also, it has been reported that self-exclusion has been tested on a poker website with at least favorable effects compared to matched control individuals, although effects were not seen in those with the most extensive gambling involvement [15]. There is limited knowledge about the characteristics of individuals who choose to self-exclude from gambling. While financial problems and the consequences of concerned significant others are mentioned among the motivators driving self-exclusion, these factors are reported to have less importance in online gamblers than in land-based gambling [16].

As expected, one potential challenge is the risk of continued gambling in other modalities than the modality from which an individual is excluded [17]. Thus, theoretically, self-exclusion from online gambling may present more challenges than a self-exclusion program related to a single gambling venue or similar land-based exclusion programs, given the large number of potential gambling sites available online. Based on this, in January 2019, the first national self-exclusion system was introduced in Sweden, allowing for self-exclusion from both land-based and online operators permitted to operate in the country, altogether a large number of gambling operators. However, little is known about which features may characterize individuals who choose to enroll in such a national multi-venue self-exclusion system. Previous literature documenting more widespread self-exclusion programs has included only casinos in a geographical setting [10], and in studies addressing online gamblers, these have tended to be related to one specific online gambling site [18–20], to selected websites in a 7-day self-exclusion program [14], or similar, to specific locations of land-based video gaming machine gambling [21]. Among the few exceptions, Pickering and co-workers reported interview data from a small sample of self-excluders in an Australian multi-venue exclusion system for land-based gambling. Here, the possibility of self-excluding from numerous gambling venues was cited as the most important advantage of the system studied, and financial consequences were among the most cited reasons for enrolling [22]. In addition, the obvious advantage of not having to exclude from every separate gambling operator, and the possibility and ease of self-excluding without entering the actual venue, have been cited as important features of a self-exclusion system [22, 23]. Thus, obviously, an easy-to-use system, available through an independent non-commercial body, may theoretically provide an attractive option to individuals who experience loss of control of their gambling.

Also, although it can be assumed that people with a problematic gambling pattern may be particularly prone to enroll, there are anecdotal reports of individuals who self-exclude in the present system due to an aversion against gambling advertising, rather than because of an actual gambling problem. Kotter and co-workers summarized that between 61 and 95% of self-excluding gamblers fulfill criteria of a diagnosis; thus, most, but not all, self-excluders have a severe gambling problem [11].

Altogether, there is lacking research describing the characteristics of individuals who self-exclude from a national self-exclusion service, and also, the knowledge about self-exclusion is more limited for online gambling than for more traditional land-based gambling activities. Therefore, previous findings may not apply to settings where a very large share of gambling—particularly problem gambling—is reported to happen online. Based on this, the present study aimed to study the characteristics of individuals enrolling in a new national multi-venue self-exclusion service within its first months of use, and factors associated with self-exclusion in a controlled model, adjusting for a number of variables believed to be associated with a problematic gambling pattern.

## Methods

The present study is an observational, nationwide general population survey, addressing individuals who are enrolled in the web panel of a market survey company in Sweden. This study originates from a survey conducted in September, 2019, and which recruited panel member from the general population to a survey addressing a number of issues related to gambling, gaming, and online behavior. Other parts and study questions related to the survey are addressed in other sub-studies.

### Setting

Gambling in Sweden is legal for individuals aged 18 years and above. Sweden has a gambling market which has been transforming in recent years. From being a previous oligopoly-based system, although with large involvement of overseas non-licensed gambling operators, the Swedish market was changed on January 1, 2019, into a license market allowing new gambling operators, under the condition that they fulfil a number of administrative and responsible gambling requirements. These include the commitment to not allow gamblers who have registered in the national self-exclusion system (‘Spelpaus’, *spelpaus.se*). Land-based electronic gambling machines and land-based casinos are still administered by the state-owned monopoly operator (Svenska spel), whereas online-based gambling activities are offered by a wide range of gambling operators. During the first month of the introduced license system (January, 2019), around 66 gambling operators were licensed by the national authority [24].

Problem gambling has been reported to affect 0.6% of the adult population from a national public health survey carried out repeatedly over time and using the well-established instrument PGSI, with problem gambling defined as a PGSI score above 7. This figure from the 2018 survey indicates an increase in problem gambling from around 0.4% in 2015 [25]. Traditionally, men have constituted the large majority of problem gamblers [26], a picture still seen in the treatment setting [27]. However, recent data have demonstrated a marked increase of women among problem gamblers [28], and in online gamblers in the present setting, problem gambling was even reported to be more likely in women than in men, with a non-significant sex difference even when controlling for a range of relevant covariates [29]. A large majority of treatment seekers report problematic online gambling, most commonly online casino and online sports betting [27].

### New self-exclusion tool—‘spelpaus.se’

One of the responsible gambling measures introduced in January, 2019, was the introduction of a nationwide self-exclusion system involving all licensed gambling operators. Previously, company-specific self-exclusion services have been applied in gambling operators licensed and permitted in Sweden, while the information about similar systems in overseas operators acting on the Swedish market prior to 2019 is more anecdotal. According to the new gambling legislation in use since January, 2019, this new self-exclusion service is mandatory for all licensed gambling operators involved in sports or horse betting, casino gambling, electronic gambling machines, both in land-based and online gambling modalities, while certain lotteries, primarily charity lotteries, as well as the smaller limited-deposit casino tables found in specific restaurants, are not covered by the self-exclusion system. During the first month of the *spelpaus* service, 19,900 individuals enrolled, and up to September, 2019, when the present study was carried out, more than 41,000 people had enrolled in the system [24].

The *spelpaus* service makes it impossible to enter licensed gambling operators for a duration of one, three, six or twelve months, depending on the choice made by the gamblers upon enrollment. The prohibition to enter licensed gambling services after self-excluding is administered by the governmental Swedish Gambling Authority, to which a rapid electronic control message is generated every time an individual attempts to enter an online gambling site or land-based operators covered by the system. In case the individual appears in the *spelpaus* system, entering any of these gambling venues or sites is prevented, and otherwise, the gambler is immediately allowed to enter. Also, *spelpaus* prohibits personalized gambling advertising to self-excluded individuals, such as gambling marketing distributed through mobile phone text messages, e-mails, and traditional mail.

### Recruitment and study participants

Participants in the present study are web panel members enrolled in the web panel of Userneeds, a market survey company operating in a number of European countries, including Sweden. Members of the Userneeds web panel typically receive offers by electronic communication to enter surveys such as markets surveys from private companies, or research or similar survey projects. In the present study, the researchers collaborated with the companies I-Mind consulting and Patient Information Broker (PIB), who gathered the data from an anonymized procedure, where Userneeds web panel members’ survey answers were sent to the authors were completely anonymized, and IP addresses of the individuals were blinded and impossible to trace back. The present type of data inclusion, from the same market survey company and using the same methodology, has been used in a number of prior studies in the present setting with a focus on gambling and online behavior in the general population [30–32].

The offer to participate was sent to web panel members in Sweden, such that individuals could voluntarily choose to enter the survey. After receiving online information about the study, the questionnaire opened only in case an individual provided informed written consent. Fulfilling a survey sent to the Userneeds web panel generates bonus merits for the respondent in Userneeds’ bonus system, which can later be translated into services or gifts provided by the company, corresponding to a value of around one Euro for each survey.

The aim was to include 2000 individuals, and recruitment was stopped after reaching that target, and also had the objective to reach a relatively representative percentage of age groups and an equal distribution of women and men. In order to achieve this, the recruitment was carried out such that each category of age and gender was actively recruited until that group’s share of the total sample was reached. From that type of recruitment method, the number of individuals who have been reached with the offer to participate cannot be established. Among included individuals, respondents in the age group 20–29 years constituted 15%, compared to 16% in the Swedish population statistics for individuals aged 15 years or older, the age group 30–39 years constituted 18% compared to 16%, the age group 40–49 years 25% compared to 15%, and the group above 50 years constituted 39%, compared to 47% in the Swedish population.

Although member accounts of web panel members are personal, in order to rule out the risk of duplicate answers, for responses deriving from the same IP address and with the same age groups, in 14 individuals with the same age group and IP address were identified as potential duplicates. For these individuals, the second survey was excluded from analyses. A total of 2117 individuals opened and started the survey, whereas 115 individuals did not complete it. The remaining 2002 individuals were included in the study.

### Measures

Socio-demographic data included age (in age groups), sex, level of education, and monthly income. Age was grouped into the categories 16–24, 25–29, 30–39, 40–49 years, and 50 years and above. Monthly income, reported in Swedish currency (SEK), was reported as being under 10,000 SEK, 10,000–15,000, 15,000–20,000, 20,000–25,000, 25,000–30,000, 30,000–35,000, 35,000–40,000, 40,000–45,000, 45,000–50,000, or 50,000 SEK and above. Level of education was defined as ‘elementary school’, ‘high school’, ‘university studies without a full degree’, ‘university studies with a full degree’, and ‘other’. In the present study, this variable was divided into ‘any post-high-school education’ or not.

Over-indebtedness (past-year history) was assessed with a dichotomous question addressing the subjective definition of over-indebtedness, as in previous research from the present setting and as recommended by the Swedish Enforcement Authority [29]. The wording of the question was ‘Have you experienced that you (or you and others living in your household) have had recurrent difficulties paying the bills during the past year?’, with the options ‘yes’, ‘no’, and ‘prefer not to answer’.

Gambling was assessed regarding past-year gambling (yes or no) on a number of common gambling types and modalities (online casino, land-based casino, online horse betting, land-based horse betting, online sports betting/odds, land-based sports betting/odds, online poker, land-based poker, land-based electronic gambling machines, online bingo, and gambling within video games).

Problem gambling was assessed using the Problem Gambling Severity Index (PGSI [33]), which aims to describe different levels of gambling-related risk and problems. It contains nine questions answered on a Likert scale, about potential past-year problems and consequences related to gambling, ranging from ‘never’ to ‘almost always’, scored from 0 to 3, with a total score of 0–27. Here, total scores were categorized into ‘no risk’ (0), ‘low risk’ (1–2), ‘moderate risk’ (3–7), and ‘problem gambling’ (8 and above), using the same instrument and the same cut-offs as in a recurrent public health survey in gambling conducted in Sweden [34].

Questions were asked about whether the respondent had ever felt a need to seek treatment for poor mental health, alcohol problems, or—defined as one item—problems related to either illicit drugs or to prescription drugs (defined as sedatives requiring prescription or strong analgesics), respectively. Also, respondents were asked about whether they smoke tobacco or use Swedish ‘snuff’ tobacco daily. All these questions had the options ‘yes’, ‘no’, and ‘prefer not to answer’.

Psychological distress was measured using the Kessler-6 [35], which includes six items describing symptoms of poor mental health during the past six months (nervousness, hopelessness, restlessness, depressed mood, feelings that everything was an effort, and worthlessness). These are rated on a Likert scale, coded from 0 (‘not at all’) to 4 ‘most of the time’ (or ‘prefer not to answer’), thus with a total score of 0–24. As previously described and validated in the literature [36, 37], a score of 13 or more was judged to represent severe psychological distress. In cases where not all six items had been responded, the cut-off between the absence and presence of severe psychological distress could be established for 35 respondents from the present items (13 points or above). In the 13 remaining cases, where four or five items were present, the median of these items was imputed in order to replace the remaining items and the cut-off was set accordingly.

The self-exclusion item was defined in the following way: ‘Since January 1st, 2019, there is a nationwide service, the *spelpaus.se*, where one can self-exclude from all legal gambling for money. Have you self-excluded using the *spelpaus.se*?’, with the options ‘yes’, ‘no’, and ‘prefer not to answer’.

### Statistical methods

Differences between respondents reporting self-exclusion and no self-exclusion were described using Chi-square tests (including the Chi-square linear-by-linear measure for age group, income categories, and gambling risk categories). The Fisher’s exact test was used whenever the number of one group was five or less. In the overall sample, a binary, non-stepwise logistic regression analysis was run, with self-exclusion (yes or no) as the dependent variable, and including potential independent correlates as independent variables; socio-demographic situation (age group expressed as a continuous variable, sex, and level of education), financial situation (monthly income, self-reported over-indebtedness), comorbidity (tobacco, alcohol, drug problems and psychological distress), as well as gambling risk category. Detailed history of gambling activities is demonstrated in Table 1 for descriptive purposes but not included in the logistic regression, due to the low absolute number of individuals with the positive outcome (self-exclusion). In the logistic regression analysis, the gambling risk variable was treated as a categorical variable, comparing each level to the ‘no-risk’ category (PGSI 0) as the category of reference. Within the sub-categories of problem gamblers and non-problem gamblers, respectively, descriptive data compared self-excluders to non-self-excluders, whereas due to the low absolute number of self-excluders, no regression analyses were carried out for these sub-group analyses.

**Table 1:** Comparison of self-excluders and non-self-excluders (including all individuals with valid answer for the self-exclusion item, *N* = 1940)

	Self-excluded (*n* = 84)	Not self-excluded (*n* = 1856)	*p* value	Missing
Male sex	58% (49)	49% (908)	0.09	2
Age			< 0.001*	0
16–19	14% (12)	2% (45)		
20–24	17% (14)	5% (95)		
25–29	17% (14)	9% (169)		
30–39	25% (21)	18% (331)		
40–49	17% (14)	25% (466)		
50 and above	11% (9)	40% (750)		
Post-high-school education	43% (36)	57% (1049)	0.01	0
Income	11% (9)	9% (171)	0.26*	0
Under 10,000				
10,000–15,000-	14% (12)	9% (169)		
15,000–20,000	7% (6)	9% (172)		
20,000–25,000	8% (7)	10% (191)		
25,000–30,000	20% (17)	16% (289)		
30,000–35,000	15% (13)	16% (294)		
35,000–40,000	7% (6)	12% (220)		
40,000–45,000	6% (5)	7% (125)		
45,000–50,000	1% (1)	3% (64)		
50,000 and above	10% (8)	9% (161)		
Past-year over-indebtedness	20% (17)	4% (80)	< 0.001	9
Need for psychological distress treatment	54% (45)	36% (662)	< 0.001	25
Severe psychological distress	24% (20)	9% (161)	< 0.001	10
Need for treatment for alcohol problems	15% (12)	4% (68)	< 0.001	8
Need for treatment for drug problems	12% (10)	2% (30)	< 0.001	8
Tobacco smoking	26% (21)	16% (294)	0.02	7
Gambling risk			< 0.001*	55
None	76% (47)	90% (1645)		
Low	3% (2)	5% (90)		
Moderate risk	6% (4)	2% (42)		
Problem gambling	15% (9)	3% (46)		
*Past-year gambling activities*				
Online casino	28% (23)	7% (136)	< 0.001	11
Physical casino	15% (12)	3% (63)	< 0.001	12
Online horse betting	19% (15)	14% (256)	0.24	13
Physical horse betting	16% (13)	11% (202)	0.13	17
Online sports betting	21% (16)	17% (310)	0.39	14
Physical sports betting	17% (14)	12% (217)	0.13	11
Online poker	13% (10)	3% (59)	< 0.001	10
Physical poker	7% (6)	3% (59)	0.04	10
Physical gambling machines	17% (14)	5% (88)	< 0.001	7
Online bingo	13% (10)	5% (90)	< 0.01	11

Chi-square analyses

*Chi-square, linear-by-linear

## Results

Among the 2002 individuals who completed the survey, sixty-two individuals preferred not to answer the question about self-exclusion. Among the remaining 1940 individuals, with a valid response to the self-exclusion item, 4% (*n* = 84) reported self-exclusion.

In the unadjusted analysis, comparing self-excluders to the remaining respondents in the full sample, self-exclusion was significantly associated with younger age, lower education, past-year over-indebtedness, severe psychological distress, tobacco use, treatment needs for psychological distress, alcohol problems and drug problems, respectively, as well as a significant association with increasing category of gambling risk (PGSI). With respect to separate gambling activities, self-exclusion was significantly more common in individuals reporting past-year gambling on online casino, land-based casino, online poker, land-based poker, land-based electronic gambling machines, and online bingo, respectively (Table 1). In logistic regression of socio-demographic and comorbidity variables, self-exclusion remained significantly associated with younger age group (OR 0.65 [0.54–0.79] for older age group) and with the highest level of PGSI level (OR 2.84 [1.10–7.37]) compared to the reference value (non-problem gambling). In contrast, a history of self-exclusion, in this adjusted model, was not associated with sex, level of education, monthly income, a history of over-indebtedness, and also unrelated to tobacco smoking, psychological distress or a history of treatment needs for alcohol or drug problems (Table 2).

**Table 2:** Logistic regression, potential correlates of self-exclusion

	OR	95% confidence interval
Male sex	1.07	0.62–1.85
Age group (older)	0.65	0.54–0.79*
Post-high-school education	0.88	0.50–1.55
Income (increasing category)	1.06	0.94–1.19
Past-year over-indebtedness	2.01	0.80–5.06
Severe psychological distress	1.38	0.65–2.91
Treatment needs for alcohol problems	1.36	0.40–4.68
Treatment need for drug problems	1.25	0.28–5.58
Daily tobacco smoking	1.16	0.59–2.29
PGSI score (reference: no-risk gambling)		
Low risk gambling	0.71	0.17–3.03
Moderate-risk gambling	2.25	0.74–6.84
Problem gambling	2.84	1.10–7.37*

All respondents with full data (*n* = 1859). Non-categorical independent variables (except for PGSI score, using no-risk gambling as reference)

*Significant association

In the subgroup of respondents with a gambling problem (moderate-risk or problem gambling), 13 out of 101 individuals with full data were self-excluded (13%). In this group, self-exclusion was significantly associated with younger age (*p* < 0.05) and severe psychological distress (*p* = 0.02, Table 3). In the subgroup without moderate-risk or problem gambling, 49 out of 1784 (3%) were self-excluded. In this group, self-exclusion was significantly associated with younger age (*p* < 0.001) and with past-year over-indebtedness (*p* < 0.001), Table 4).

**Table 3:** Problem gamblers (*n* = 101)

	Self-excluded (*n* = 13)	Not self-excluded (*n* = 88)	*p* value	Missing
Male sex	77% (10)	60% (53)	0.25	0
Age			0.05*/**	0
16–19	8% (1)	7% (6)		
20–24	38% (5)	14% (12)		
25–29	15% (2)	15% (13)		
30–39	23% (3)	28% (25)		
40–49	15% (2)	26% (23)		
50 and above	0% (0)	10% (9)		
Post-high-school education	54% (7)	44% (39)	0.52	0
Income			0.53**	0
Under 10,000	8% (1)	14% (12)		
10,000–15,000	23% (3)	13% (11)		
15,000–20,000	15% (2)	9% (8)		
20,000–25,000	8% (1)	14% (12)		
25,000–30,000	31% (4)	14% (12)		
30,000–35,000	8% (1)	18% (16)		
35,000–40,000	0% (0)	10% (9)		
40,000–45,000	0% (0)	3% (3)		
45,000–50,000	0% (0)	1% (1)		
50,000 and above	8% (1)	5% (4)		
Over-indebtedness	23% (3)	14% (12)	0.41***	3
Need for psychological distress treatment	62% (8)	38% (33)	0.11	2
Severe psychological distress	46% (6)	18% (16)	0.02	1
Need for treatment for alcohol problems	23% (3)	13% (11)	0.39***	3
Need for treatment for drug problems	23% (3)	14% (12)	0.41***	1
Daily tobacco smoking	42% (5)	29% (25)	0.36	2
*Past-year gambling activities*				
Online casino	42% (5)	38% (32)	0.79	4
Physical casino	23% (3)	14% (12)	0.41***	2
Online horse betting	23% (3)	26% (22)	1.00***	3
Physical horse betting	15% (2)	20% (17)	1.00***	5
Online sports betting	36% (4)	43% (37)	0.76***	4
Physical sports betting	17% (2)	26% (22)	0.72***	3
Online poker	15% (2)	18% (15)	1.00***	3
Physical poker	8% (1)	12% (10)	1.00***	2
Physical gambling machines	23% (3)	20% (17)	0.72***	1
Online bingo	15% (2)	30% (26)	0.34***	1
Problem gambling (PGSI > 7)	69% (9)	52% (46)	0.37***	0

Comparison of self-excluders and non-self-excluders. Chi-square analyses

* < 0.05, rounded off to 0.05

**Chi-square, linear-by-linear

***Fisher’s exact test

**Table 4:** Non-problem gamblers (*n* = 1784)

	Self-excluded (*n* = 49)	Not self-excluded (*n* = 1735)	*p* value	Missing
Male sex	43% (21)	48% (828)	0.50	2
Age			< 0.001*	0
16–19	10% (5)	2% (35)		
20–24	6% (3)	4% (75)		
25–29	16% (8)	9% (152)		
30–39	27% (13)	17% (302)		
40–49	22% (11)	25% (434)		
50 and above	18% (9)	42% (737)		
Post-high-school education	49% (24)	57% (993)	0.25	0
Income			0.56*	0
Under 10,000	10% (5)	9% (157)		
10,000–15,000-	12% (6)	9% (153)		
15,000–20,000	8% (4)	9% (159)		
20,000–25,000	10% (5)	10% (177)		
25,000–30,000	16% (8)	16% (272)		
30,000–35,000	12% (6)	16% (273)		
35,000–40,000	12% (6)	12% (208)		
40,000–45,000	8% (4)	7% (120)		
45,000–50,000	2% (1)	4% (63)		
50,000 and above	8% (4)	9% (153)		
Over-indebtedness	12% (6)	3% (57)	< 0.001	5
Need for psychological distress	43% (21)	36% (615)	0.31	20
Severe psychological distress	10% (5)	8% (133)	0.52	8
Need for treatment for alcohol problems	4% (2)	3% (49)	0.65**	2
Need for treatment for drug problems	4% (2)	1% (13)	0.06**	2
Daily tobacco smoking	16% (8)	15% (260)	0.80	3
*Past-year gambling activities*				
Online casino	8% (4)	5% (90)	0.32**	4
Physical casino	2% (1)	2% (40)	1.00**	4
Online horse betting	6% (3)	13% (221)	0.27**	5
Physical horse betting	4% (2)	10% (178)	0.22**	4
Online sports betting	6% (3)	15% (259)	0.10**	4
Physical sports betting	2% (1)	11% (187)	0.05**/***	4
Online poker	0% (0)	2% (33)	1.00**	2
Physical poker	2% (1)	2% (43)	1.00**	3
Physical gambling machines	2% (1)	4% (64)	1.00**	2
Online bingo	8% (4)	3% (52)	0.06**	4

Comparison of self-excluders and non-self-excluders. Chi-square analyses

*Chi-square, linear-by-linear

**Fisher’s exact test

***Above cut-off 0.05

## Discussion

The present study was, to the best of the authors’ knowledge, one of the few studies examining the characteristics of people who choose to join a nationwide, multi-venue gambling self-exclusion system. In particular, the study provides a comparison of self-excluders to non-self-excluders in the general population, with respect to the first few months after this system was introduced. As expected, self-excluders were more likely to be problem gamblers, although the majority of them did not screen positive for a past-year problem gambling, and also, problem gamblers were found among respondents who had not chosen to self-exclude. While self-excluders presented more extensive past-year gambling behavior, few demographic variables separated them from other respondents, and the main variables characterizing self-excluders were younger age, and the higher likelihood of problem gambling. Among clients without past-year problem gambling or moderate risk gambling, over-indebtedness was associated with self-exclusion.

In the present study, both in the unadjusted and adjusted analysis, self-exclusion was unrelated to sex. Motka and co-workers summarized a number of studies related to self-exclusion. Here, all studies assessing online gamblers reported that a majority of self-excluders were men, ranging from 69 to 95% male self-excluders. In contrast, however, between 45 and 72% of those self-excluding from land-based gambling were male, whereas the sex proportion also appeared to differ by geographical setting [16]. In the present study, among self-excluders, 58% were men, therefore within the range of the studies presenting the sex distribution in self-excluders, but lower than previous studies reporting self-exclusion in online gambling specifically.

The lack of a sex difference in the present study may seem to be in contrast with the traditionally male majority reported in samples of problem gamblers [26, 38–41], although recent reports from the present geographical setting may seem to be a recent exception; recent general population data [28], and survey data in online gamblers [29], demonstrate that women may be as likely as men to report problem gambling as measured with the PGSI. The results of the present study corroborate with the findings of Dragicevic and co-workers, who concluded that among online gamblers, male sex was not overrepresented in self-excluders, and previous data showing a male majority may be explained by the higher gambling prevalence in men [42].

Traditionally, in the literature describing general population samples or clinical samples of problem gamblers, men are markedly more common than women. In recent years, from the present setting, however, it has been reported that female sex is even more common than male sex in problematic online gamblers [29]. In the present setting, online gambling has been rapidly increasing in recent years, and constitutes a large majority of the gambling types reported by treatment-seeking problem gamblers [27] and a large majority of gambling-related television advertisements [29]. Thus, although the self-exclusion system discussed here involves both land-based and online gambling in a large range of gambling types, the population addressed is more likely to be online gamblers than in settings where online gambling still is less predominating than in the present setting.

Here, self-excluders were significantly younger than other respondents, both in the unadjusted analysis, and when controlling for problem gambling and other variables. This is consistent with the study of Dragicevic and colleagues, who reported that self-excluders were younger and had experience more gambling losses, compared to a control group [42]. Likewise, although subjective indebtedness was not associated with self-exclusion in the subgroup of problem gamblers in the present study, it was strongly associated with self-exclusion among non-problem gamblers, still indicating that financial situation may increase the likelihood of a person self-excluding from gambling.

Self-exclusion was markedly more common in problem gamblers, and in the logistic regression analysis, along with younger age, the most severe level of gambling was the only variable significantly and independently associated with self-exclusion. While this is far from surprising, the relatively low level of past-year problem gambling in the present sample of self-excluders may merit further research. Here, only around one fifth of respondents were either moderate-risk or problem gamblers. In a study by Ladouceur, 73% of casino self-excluders fulfilled criteria of a gambling disorder [3], and in another study of casino self-excluders from the same research group, virtually all self-excluders fulfilled diagnostic criteria [2]. In Hayer and Meyer’s study of casino self-excluders, only around 52% fulfilled diagnostic criteria, whereas around three out of four self-excluders were at least problem gamblers [43]. In Nelson’s study of casino self-excluders, 79%, although assessed retrospectively, met the criteria of a probable disorder [10].

Here, it should be borne in mind that the present study was not a survey addressing self-excluders specifically, but rather examined gambling self-exclusion along with a number of other variables in a survey addressing the general population. Still, however, it may seem surprising that problem gambling did not more distinctly separate self-excluders from non-excluders. In previous studies, summarized in the review of Motka et al. [16] aims of self-excluding have been summarized as being caused by problematic gambling and its consequences, such as relationship or financial consequences. There is little evidence describing the mechanisms behind self-excluding from gambling in individuals who do not fulfill criteria of problem gambling. The type of national self-exclusion service, as assessed above after its introduction in Sweden in January, 2019, is previously not discussed in the scientific literature. Thus, the present study may be the first study indicating the socio-demographic and other characteristics of people who self-exclude during the first months of such a new system, and more research is needed in order to understand the mechanisms behind self-excluding in current non-problem gamblers.

However, one factor which may elucidate this is that self-reported, subjective over-indebtedness was markedly more common in non-problem gamblers reporting self-exclusion, than in non-problem gamblers who had not excluded themselves from gambling. Thus, individuals who do not currently screen positive for problem gambling may choose self-exclusion due to financial problems, due to a previous gambling problem in order to prevent themselves from relapse, or even in order to cut back on household expenditures. Also, it should be noted that the present type of self-exclusion system, where one can self-exclude without any physical or online contact with any gambling operator, more easily facilitates this kind of self-excluding behavior. Also, this may therefore also include currently non-problematic gamblers or non-gamblers, for example in a person who perceives herself/himself to have a high risk of relapsing into a previously problematic behavior, and who wishes to prevent such a relapse without entering a gambling operator’s site or venue.

One particular feature of self-exclusion systems, including in the present setting, is that the self-exclusion service may theoretically be used not only for self-exclusion from gambling, but also in order to avoid exposure to direct marketing by gambling operators. The self-exclusion through the *spelpaus* system involves the prohibition of direct advertising such as text messages or e-mails to the self-excluded gamblers, i.e. the type of advertising that has been described to be effective in stimulating gambling [44]. It is unknown to what extent this may contribute to people’s willingness to self-exclude from gambling, as this also makes gambling in most venues impossible during the self-exclusion period. However, gambling advertising has expanded significantly in the present setting in recent years, and it has been reported to be particularly disturbing to individuals with a gambling problem [45, 46]. Gambling advertising in the present setting is clearly skewed towards a large share of advertisements promoting online casino games [47], which is also the gambling modality most commonly reported by treatment-seeking patients in the setting studied here [27]. One can only speculate about whether people may self-exclude only because of having concerns about gambling advertising without having an actual gambling problem, but such a reason for self-excluding cannot be excluded. In particular, due to the large expansion of advertising in recent years, there has been a general political concern over gambling advertising [48], and which is likely to reflect the general public’s attitude to this type of advertising.

The present study has implications for further research and for stakeholders working in prevention and harm reduction related to gambling problems. For example, a majority of past-year problem or moderate-risk gamblers in the present study did not report a history of self-excluding even from a national, multi-venue exclusion system. Thus, although in a highly available self-exclusion system, a large percentage of the target group may not choose that measure to control their own gambling, such that further promotion of this option may be needed. Also, this may require further motivational approaches from authorities consulted by people at risk of gambling problems but who may not be aware of—or may not have chosen—self-exclusion. This may apply to advisors in consumer credit situations seen by people with increasing indebtedness, and to counselors in social services, mental health treatment, or in enforcement authorities in their contact with over-indebted individuals. Indeed, from the present findings, it can be suspected that among the numbers of individuals self-excluded from gambling, a substantial proportion may not be problem gamblers, such that the sole number of individuals registered in the system may not give the full picture of how such a system covers the needs among the people actually at risk. Likewise, it should be emphasized that gambling operators are likely to be the first source of information of a loss-of-control pattern in an individual’s gambling pattern, and systems should be developed for gambling operators to promote and facilitate self-exclusion for gamblers demonstrating signs of a problem behavior. As a large percentage of problem gamblers in the present study were not self-excluded, there may be potential to expand self-exclusion significantly through such responsible gambling initiatives operating from within the gambling industry.

### Limitations

There are potential limitations in the present study, largely depending on the fact that study data rely on self-report, and that among web panel members with a greater interest in gambling-related issues may have been more prone to respond than others, as the survey was presented as related to online and gambling behavior. Therefore, it is highly likely that more gamblers, and more high-risk gamblers, could be prone to accept the present survey, compared to the general population. According to the data from the Swedish Gambling Authority [24], around 41,000 individuals had self-excluded until the present study was carried out, reflecting roughly half a percent of the country’s adult population. Thus, in the present study, where 4% of those who gave a ‘yes’ or ‘no’ answer to the self-exclusion question endorsed this item, is likely due to the survey attracting people with more extensive gambling habits. Also, it cannot be excluded that people who enroll with a web panel of a market survey company, such as the one used here, have other online habits and other gambling habits than their counterparts in the general population. However, still, it should be possible to examine differences between self-excluders and others, but findings should be seen in light of this and interpreted with caution.

Also, despite the higher rates of self-excluders in the present dataset, as the absolute number of self-excluders is low, regression analyses could not be carried out for the sub-groups of problem gamblers and non-problem gamblers. Also, specific gambling activities could not be included in the overall logistic regression model with the self-exclusion as their dependent variable, also due to the low number of respondents. While this is a limitation, future studies of self-excluders in the general population may need to include larger samples of respondents from the general public, in order to allow for more elaborate statistical predictions. However, the present data still demonstrate findings of relevance to the description of how self-excluders may differ from non-self-excluders in the general population, and propose patterns of individuals characteristics shown in these two categories, respectively.

Another limitation is that the study sample included age groups from 16 years of age (with 64 individuals in the age group 16–24 years). Thus, in the present sub-study of factors associated with self-exclusion from gambling, a lower number of individuals could be 16 or 17 years old, although the legal gambling age, and the age limit for enrollment in the self-exclusion system, are 18 years of age. In the final results, younger age was associated with self-exclusion, and given the small number of individuals potentially below 18 years of age, this association would rather have been underestimated, as a smaller number of respondents may not have the legal possibility self-exclude. Although this number of individuals is likely very low, a second logistic regression analysis was run, excluding the youngest age group (including a final number of 1814 individuals in the regression model), where the same variables were associated with self-exclusion, i.e. younger age group and the highest PGSI level (problem gambling). Thus, the present limitation must be considered to have a very limited impact on the study findings.

## Conclusions

After the introduction of a novel nationwide system of self-exclusion from gambling, enrollment into such a system appears to be associated with younger age and, not surprisingly, with problem gambling. However, self-exclusion in this type of system may also apply to broader groups than only individuals who screen positive for a recent gambling problem. However, several potentially high-risk-oriented gambling activities were more common in self-excluders than among others. Sex differences between self-excluders and other individuals in the general population were small and non-significant in the present study.

## Data Availability

Study data can be obtained from the authors, after review by the ethics board.

## References

[1] Calado F, Griffiths M (2016). Problem gambling worldwide: an update and systematic review of empirical research (2000–2015). J Behav Addict..

[2] Ladouceur R, Jacques C, Giroux I, Ferland F, Leblond J (2000). Analysis of a casino’s self-exclusion program. J Gambl Stud.

[3] Ladouceur R, Sylvain C, Gosselin P (2007). Self-exclusion program: a longitudinal evaluation study. J Gambl Stud.

[4] Napolitano F (2003). The self-exclusion program: legal and clinical considerations. J Gambl Stud.

[5] Matheson FI, Hamilton-Wright S, Kryszajtys DT, Wiese JL, Cadel L, Ziegler C (2019). The use of self-management strategies for problem gambling: a scoping review. BMC Publ Health.

[6] McMahon N, Thomson K, Kaner E, Bambra C (2019). Effects of prevention and harm reduction interventions on gambling behaviours and gambling related harm: an umbrella review. Addict Behav.

[7] Abbot MW (2019). Self-directed interventions for gambling disorder. Curr Opin Psychiatry.

[8] Nower L, Blaszczynski A (2006). Characteristics and gender differences among self-excluded casino problem gamblers: Missouri data. J Gambl Stud.

[9] Tremblay N, Boutin C, Ladouceur R (2008). Improved self-exclusion programs: preliminary results. J Gambl Stud.

[10] Nelson SE, Kleschinsky JH, LaBrie RA, Kaplan S, Shaffer HJ (2010). One decade of self exclusion: Missouri casino self-excluders four to ten years after enrollment. J Gambl Stud.

[11] Kotter R, Kräplin A, Pittig A, Bühringer G (2019). A systematic review of land-based self-exclusion programs: demographics, gambling behavior, gambling problems, mental symptoms, and mental health. J Gambl Stud.

[12] Hing N, Russell AM, Gainsbury SM, Blaszczynski A (2015). Characteristics and help-seeking behaviors of Internet gamblers based on most problematic mode of gambling. J Med Internet Res.

[13] Gainsbury SM, Angus DJ, Procter L, Blaszczynski A. Use of consumer protection tools on internet gambling sites: customer perceptions, motivators, and barriers to use. J Gambl Stud. 2019 [e-pub ahead of print] 10.1007/s10899-019-09859-8 31119509

[14] Caillon J, Grall-Bronnec M, Perrot B, Leboucher J, Donnio Y, Romo L (2019). Effectiveness of at-risk gamblers’ temporary self-exclusion from internet gambling sites. J Gambl Stud.

[15] Luquiens A, Dugravot A, Panjo H, Benyamina A, Gaïffas S, Bacry E (2019). Self-exclusion among online poker gamblers: effects on expenditure in time and money as compared to matched controls. Int J Environ Res Publ Health.

[16] Motka F, Grüne B, Sleczka P, Braun B, Örnberg JC, Kraus L (2018). Who uses self-exclusion to regulate problem gambling? A systematic literature review. J Behav Addict.

[17] McCormick AV, Cohen IM, Davies G (2018). Differential effects of formal and informal gambling on symptoms of problem gambling during voluntary self-exclusion. J Gambl Stud.

[18] Xuan Z, Shaffer H (2009). How do gamblers end gambling: longitudinal analysis of Internet gambling behaviors prior to account closure due to gambling related problems. J Gambl Stud.

[19] Braverman J, Shaffer HJ (2012). How do gamblers start gambling: identifying behavioural markers for high-risk internet gambling. Eur J Publ Health.

[20] Hayer T, Meyer G (2011). Internet self-exclusion: characteristics of self-excluded gamblers and preliminary evidence for its effectiveness. Int J Ment Health Addict.

[21] Townshend P (2007). Self-exclusion in a public health environment: an effective treatment option in New Zealand. Int J Ment Health Addict.

[22] Pickering D, Blaszczynski A, Gainsbury SM (2018). Multi-venue self-exclusion for gambling disorders: a retrospective process investigation. J Gambl Iss.

[23] Hing N, Nuske E (2012). The self-exclusion experience for problem gamblers in south Australia. Austr Soc Work.

[24] Swedish Gambling Authority. Swedish Gambling Authority, 2019. https://www.spelinspektionen.se/spelproblem1/spelpaus/spelpaus-statistik/. Accessed 25 Jan 2020).

[25] Public Health Agency of Sweden, 2019. Allvarliga spelproblem ökar bland kvinnor [Severe gambling problems increase in women]. https://www.folkhalsomyndigheten.se/nyheter-och-press/nyhetsarkiv/2019/april/allvarliga-spelproblem-okar-bland-kvinnor/. Accessed 17 Sept 2020 (in Swedish).

[26] Abbott MW, Romild U, Volberg RA (2018). The prevalence, incidence, and gender and age-specific incidence of problem gambling: results of the Swedish longitudinal gambling study (Swelogs). Addiction.

[27] Håkansson A, Mårdhed E, Zaar M (2017). Who seeks treatment when medicine opens the door to gambling disorder patients—psychiatric co-morbidity and heavy predominance of online gambling. Front Psychiatry.

[28] BBC. Sweden female gambling addicts outnumber men for first time. https://www.bbc.com/news/world-europe-47814630. BBC, 2019. Accessed 29 Oct 2019.

[29] Håkansson A, Widinghoff C (2020). Indebtedness and problem gambling in a general population sample of online gamblers. Front Psychiatry.

[30] Karlsson J, Broman N, Håkansson A (2019). Associations between problem gambling, gaming, and internet use: a cross-sectional population survey. J Addict.

[31] Håkansson A, Ford M (2019). The general population’s view on where to seek treatment for gambling disorder—a general population survey. Psychol Res Behav Manag.

[32] Ford M, Håkansson A (2020). Problem gambling, associations with comorbid health conditions, substance use, and behavioural addictions: opportunities for pathways to treatment. PLoS ONE.

[33] Wynne H, Ferris J. The Canadian Problem Gambling Index: Final Report.Ottawa: Canadian Centre on Substance Abuse (CCSA), 2001.

[34] Public Health Agency of Sweden. Swelogs. https://www.folkhalsomyndigheten.se/the-public-health-agency-of-sweden/living-conditions-and-lifestyle/alcohol-narcotics-doping-tobacco-and-gambling/gambling/swelogs/ Public Health Agency of Sweden, 2015. Accessed 25 Jan 2020.

[35] Furukawa TA, Kessler RC, Slade T, Andrews G (2003). The performance of the K6 and K10 screening scales for psychological distress in the Australian National Survey of Mental Health and Well-Being. Psychol Med.

[36] Kessler RC, Barker PR, Colpe LJ, Epstein JF, Gfroerer JC, Hiripi E (2003). Screening for serious mental illness in the general population. Arch Gen Psychiatry.

[37] Prochaska JJ, Sung HY, Max W, Shi Y, Ong M (2012). Validity study of the K6 scale as a measure of moderate mental distress based on mental health treatment need and utilization. Int J Methods Psychiatr Res.

[38] Blanco C, Hasin DS, Petry N, Stinson FS, Grant BF (2006). Sex differences in subclinical and DSM-IV pathological gambling: results from the National Epidemiologic Survey on Alcohol and Related Conditions. Psychol Med.

[39] Ekholm O, Eiberg S, Davidsen M, Holst M, Larsen CV, Juel K (2014). The prevalence of problem gambling in Denmark in 2005 and 2010: a sociodemographic and socioeconomic characterization. J Gambl Stud.

[40] Håkansson A, Karlsson A, Widinghoff C (2018). Primary and secondary diagnoses of gambling disorder and psychiatric comorbidity in the Swedish health care system—a nationwide register study. Front Psychiatry.

[41] Carneiro E, Tavares H, Sanches M, Pinsky I, Caetano R, Zaleski M (2019). Gender differences in gambling exposure and at-risk gambling behavior. J Gambl Stud..

[42] Dragicevic S, Percy C, Kudic A, Parke J (2015). A descriptive analysis of demographic and behavioral data from internet gamblers and those who self-exclude from online gambling platforms. J Gambl Stud.

[43] Hayer T, Meyer G (2011). Self-exclusion as a harm minimization strategy: evidence for the casino sector from selected European countries. J Gambl Stud.

[44] Russell AMT, Hing N, Browne M, Rawat V (2018). Are direct messages (texts and emails) from wagering operators associated with betting intention and behavior? An ecological momentary assessment study. J Behav Addict.

[45] Hanss D, Mentzoni RA, Grifftiths MD, Pallesen S (2015). The impact of gambling advertising: problem gamblers report stronger impacts on involvement, knowledge, and awareness than recreational gamblers. Psychol Addict Behav.

[46] Binde P, Romild U (2019). Self-reported negative influence of gambling advertising in a Swedish population-based sample. J Gambl Stud.

[47] Håkansson A, Widinghoff C (2019). Television gambling advertisements: extent and content of gambling advertisements with a focus on potential high-risk commercial messages. Addict Behav Rep.

[48] Radio Sweden. Government looking to limit gambling ads. https://sverigesradio.se/sida/artikel.aspx?programid=2054&artikel=7205039. Radio Sweden, 2019. Accessed 25 Jan 2020.

